# Mathematics applied to the climate system: outstanding challenges and recent progress

**DOI:** 10.1098/rsta.2012.0518

**Published:** 2013-05-28

**Authors:** Paul D. Williams, Michael J. P. Cullen, Michael K. Davey, John M. Huthnance

**Affiliations:** 1 Department of Meteorology, University of Reading, Earley Gate, Reading RG6 6BB, UK; 2 Met Office, FitzRoy Road, Exeter, Devon EX1 3PB, UK; 3 National Oceanography Centre, 6 Brownlow Street, Liverpool L3 5DA, UK; 4 Department of Applied Mathematics and Theoretical Physics, University of Cambridge, Wilberforce Road, Cambridge CB3 0WA, UK

**Keywords:** climate change, applied mathematics, model evaluation, conceptual models, general circulation models, statistical frameworks

## Abstract

The societal need for reliable climate predictions and a proper assessment of their uncertainties is pressing. Uncertainties arise not only from initial conditions and forcing scenarios, but also from model formulation. Here, we identify and document three broad classes of problems, each representing what we regard to be an outstanding challenge in the area of mathematics applied to the climate system. First, there is the problem of the development and evaluation of simple physically based models of the global climate. Second, there is the problem of the development and evaluation of the components of complex models such as general circulation models. Third, there is the problem of the development and evaluation of appropriate statistical frameworks. We discuss these problems in turn, emphasizing the recent progress made by the papers presented in this Theme Issue. Many pressing challenges in climate science require closer collaboration between climate scientists, mathematicians and statisticians. We hope the papers contained in this Theme Issue will act as inspiration for such collaborations and for setting future research directions.

## 1. Introduction

The societal need for reliable climate predictions and a proper assessment of their uncertainties is pressing. However, the climate system is complex with a multitude of spatial and temporal scales. Although the governing equations of the underlying fluid dynamics are known essentially exactly in the continuum limit, an accurate resolution of their solutions down to the smallest energized scales is well beyond the capacity of any computers that are available now or foreseeable in the future. In addition, many climate-critical processes such as convection and clouds are represented only approximately in climate simulations, and perhaps always will be. Such processes may not be amenable to a representation by physically based deterministic equations and may in fact be inherently stochastic. For all these reasons, uncertainties in climate predictions arise not only from uncertainties in initial conditions and forcing scenarios (including future emissions of volcanic aerosols and greenhouse gases), but also from inherent uncertainties in model formulation.

The ability to make predictions of the climate system as a whole is hindered because of a lack of accepted physical principles that control the overall behaviour, such as the global-mean temperature. Without such principles, it is impossible to estimate the error of a prediction made with an inevitably imperfect model over a long evolution time. Nevertheless, reliable climate predictions are needed urgently, not least because they influence the risk assessments used by policy makers. Therefore, it is essential that climate predictions sample all the possible sources of uncertainty and encompass all the possible outcomes, as discussed by Collins *et al.* [1]. Recent Theme Issues of this journal relevant to this topic include those compiled by Collins [2], Palmer & Williams [3], Thompson [4], Nikiforakis [5], Palmer & Hardaker [6] and Thompson & Sieber [7].

From the perspective of an applied mathematician, there are two approaches to studying the climate system. The first approach uses simple, conceptual models with only a few degrees of freedom. These models are designed to capture the observed relationships between components relevant to a particular phenomenon, such as time series of key integrated variables like global surface air temperature and global atmospheric composition, without attempting to represent the full three-dimensional evolution. The simplicity of these models makes it easier to focus on relationships between selected processes and to explore parameter dependencies. The simplicity often makes simple models amenable to analytic progress. However, the simplicity also limits the usefulness of simple models as tools for quantitative prediction.

The second approach is to use general circulation models (GCMs), which contain a wide range of physical processes represented through millions of degrees of freedom. These models are designed to capture the full spatial and temporal evolution of the atmosphere–ocean–land system. Also in this category are Earth-system models and Earth-system models of intermediate complexity, which include comprehensive, interactive representations of the cryosphere and the global carbon cycle. Similarly, large-eddy simulations (LESs) of the atmospheric flow are designed to represent interactions spanning several time and space scales. The complexity of these models makes them useful as tools for quantitative prediction, but heavy supercomputing infrastructure is required and analytic progress is impossible.

Simple and complex models have different strengths and weaknesses. A major weakness of simple models, particularly those based largely on statistical relationships, is the problem of establishing a rigorous physical basis for them, given that many important processes are neglected. Recent advances in statistics allow such models to be fitted to observed data in a more dynamical framework, making due allowances for their limitations. However, that this can be done carries no guarantee that simple models will respond correctly to perturbations. Consistency with observed data must be regarded as a necessary but not sufficient condition for demonstrating skill. The quantitative ability of simple models to capture future forced changes to the climate is unproven by historic data-fitting exercises. It is essential to build up a physical understanding of the reasons for changes, so that the simple models can be evaluated.

A major difficulty faced by all models, particularly GCMs, is the huge range of length and time scales for key elements of the climate system. This disparity makes direct numerical simulation very difficult. For example, the time scales for changes to the deep ocean circulation and major ice sheets are centuries, whereas the time scale for the evolution of weather systems in the atmosphere is only hours. In addition, finite computing resources mean that the fastest and smallest scales cannot be simulated explicitly with global coverage.

Because of the transfer of information between resolved and unresolved scales in GCMs, the equations for the resolved scales cannot be closed exactly. The role of unresolved scales in climate simulation has been reviewed by Williams [8]. Parametrization schemes have been developed to close the equations approximately, but the parameters in them are often poorly constrained by theory and observations. While the effects of different schemes and parameter options are relatively straightforward to explore in simpler models, the computational expense of GCMs means that such investigation is limited in full climate simulations. The tactic of parameter variation within GCMs is commonly adopted to represent in part the associated uncertainty, and mathematical methods help to inform the selection strategy. In a similar vein, stochastic terms are widely used to represent unresolved processes in GCMs, with development aided by simpler models. Both approaches have been adopted in operational forecast systems, and a recent example is given by Arribas *et al.* [9].

It is important to note that, although simple and complex models have different strengths and weaknesses, neither of these model types is free from fundamental limitations. It is essential to pursue both approaches simultaneously, as a consistency check and to obtain the maximum amount of information. Indeed, the comparison between predictions from simple and complex models often advances our understanding.

Motivated by the importance of this topic, the Institute of Mathematics and its Applications held a Conference on the Mathematics of the Climate System at the University of Reading, UK, over the period 13–15 September 2011. Many, but not all, of the papers published in this Theme Issue are based on work presented at the conference. The scientific organizing committee for the meeting consisted of the editors of this Theme Issue, together with Colin Cotter (Imperial College London), Christopher Ferro (University of Exeter) and David Stainforth (London School of Economics and Political Science). A brief meeting report was provided by Williams *et al.* [10].

This introductory paper to the Theme Issue aims to survey what its authors regard as the three broad outstanding challenges in the field (§2). The article then describes the recent progress that has been made in tackling each of the three challenges, by reference to papers published in the Theme Issue (§3). Finally, the article suggests some future research directions (§4).

## 2. Outstanding challenges

In this section, we would like to identify and document three broad classes of problems. Each of the problems represents what we regard as an outstanding challenge in the area of mathematics applied to the climate system. Any such list will necessarily be subjective and open to criticism, but we hope it will serve as a useful starting point for discussions.

First, there is the problem of the development and evaluation of simple physically based models of the global climate. This problem requires physical understanding, and ensuring that the models reflect it. Open questions pertinent to this problem include the following. Should simple models be deterministic or stochastic? Is it important whether or not simple models satisfy conservation laws? How should simple models be evaluated when confronted with observational data? Are the multiple equilibrium states that are often exhibited by simple models real or merely artefacts of the simplification?

Second, there is the problem of the development and evaluation of the components of complex models such as GCMs and of the interactions between them. Again, this problem requires physical understanding, and ensuring that the models and their components reflect it. Open questions pertinent to this problem include the following. How should the equations for the resolved scales be closed? Are stochastic closure schemes better than deterministic closure schemes? Which aspects are crucial for obtaining the correct large-scale flow and variability? How should the different components be coupled?

A related problem is the generation of appropriate initial conditions, which are needed when GCMs are used to analyse and predict weather and climate features on scales of hours to seasons and beyond. Indeed, natural variability on decadal scales—not properly regarded as climate change—is a particular problem for climate prediction. The interaction between oceans and the atmosphere is important, but disparate time scales make the coupled problem of initialization and data assimilation particularly difficult. Some recent efforts are summarized by Balmaseda *et al.* [11]. Investigations with innovative mathematical methods and a hierarchy of models are needed.

Third, there is the problem of the development and evaluation of appropriate statistical frameworks. These frameworks are needed to create credible and reliable probability distributions of real-world observables from ensembles of climate predictions. The ensembles here could be multi-model ensembles, multi-parameter ensembles, ensembles forced by different greenhouse gas scenarios, or initial-condition ensembles. Such frameworks are required in order to make valid inference from ensemble climate projections with imperfect models. Open questions pertinent to this problem include the following. How should the different ensemble members be weighted? What level of detail is needed from climate models if they are to be useful tools for impact studies?

## 3. Recent progress

In this section, we deal with each of the above three problems in turn, emphasizing the contribution made by the papers in this Theme Issue.

### (a) Development and evaluation of simple models

Four papers deal with the development and evaluation of simple models. A necessary (but not sufficient) aspect of the evaluation of simple models is to test their predictions by comparison with observed data. Kwasniok [12] does this by matching palaeo-climate records with different stochastic dynamical systems, representing different dynamical mechanisms and modelling approaches. The particular focus is on simulating the observed glacial climate transitions. A stochastic model with a relaxation operator is formulated, and the system parameters and noise levels are estimated from ice-core data. This formulation is compared with the more traditional model of noise-driven motion in a bi-stable potential. Statistical properties determined by long integrations of the models provide further useful points of comparison.

A crucial aspect of improving simple models may be to make them stochastic. Therefore, a particular focus is on testing stochastic parametrizations compared with traditional, deterministic parametrizations. Arnold *et al.* [13] do this using the Lorenz [14] model. Integrations of the full model are taken as truth. Stochastic parametrizations are developed that allow the behaviour of the full model to be approximated by a truncated version of the same model. In particular, the skill of ensemble forecasts in representing the model uncertainty is assessed, and stochastic parametrizations are found to perform better than deterministic parametrizations.

The stability properties of simple models, unlike complex models, may be investigated analytically. Sudakov & Vakulenko [15] do this by extending a classical radiative-balance model to include representation of the radiative effects of greenhouse gas emissions. It is found via an asymptotic approach that the emissions can generate instabilities in the model climate system. In particular, for sufficiently high emissions, a tipping point is reached at which the system develops multiple equilibria.

Dynamical systems theory offers many tools for advanced analysis of simple models. One such tool is the unstable periodic orbit expansion procedure. Gritsun [16] applies this procedure to the barotropic vorticity equation on the sphere, which is a simple representation of atmospheric fluid flow. The probability measure generated by this chaotic dynamical system is approximated as a weighted sum over unstable periodic orbits. It is shown that the circulation regimes of the barotropic vorticity equation can be explained in terms of the diagnosed orbits.

### (b) Development and evaluation of complex models

Four papers deal with the development and evaluation of complex models. Extreme weather events, including strong winds, are known to cause substantial societal and economic damage. Franzke [17] examines extreme winds in archived operational analysis data obtained from a GCM with observations assimilated. He finds that strong wind events over Europe are related to a particular large-scale atmospheric circulation pattern in the North Atlantic. Furthermore, a statistical analysis of the extreme wind speeds shows that they are not independent, but occur in bunches with marked clustering. A good GCM should also display this clustering when run without data assimilation. Therefore, the findings of this paper could be used as a benchmark to evaluate the extreme weather performance of GCMs.

An important aspect of climate dynamics is the influence of the stratosphere on the troposphere. The processes for this influence are not yet well understood, as discussed in the review paper by Gerber *et al.* [18]. Cullen & Ngan [19] study the influence of the stratosphere on the troposphere by showing that, in archived operational analyses obtained from a GCM with observations assimilated, the ratio between the horizontal and vertical scales of anomalous circulations agrees with dynamical theory. This result yields a mechanism through which stratospheric fluctuations may affect tropospheric variability, by influencing the vertical extent of tropospheric developments.

A challenge for improving GCMs is to understand the coupling between the sub-grid parametrizations and the resolved dynamics. It is arguable that such coupling has received much less research effort relative to the separate development of the dynamical core and parametrizations. Beare & Cullen [20] investigate this for the case of the atmospheric boundary-layer parametrization. They formulate a diagnostic equation for the circulation forced by the boundary layer. The diagnostic equation results from a comprehensive formulation of the momentum and thermodynamic balances and offers new insights into the interplay between the dynamics and the parametrization.

A crucial topic is the development of improved formulations of GCMs, and particularly an improved representation of unresolved processes, such as deep convection and clouds. Dorrestijn *et al.* [21] construct a sub-grid model of deep convection using data derived from LESs. Transitions between different cloud states are modelled with Markov chains, and the variability and spatial distributions of cloud types are found to become more accurate when local spatial coupling is introduced to the Markov chains.

### (c) Development and evaluation of statistical frameworks

Four papers deal with the development and evaluation of statistical frameworks. A crucial aspect is the use of stochastic data assimilation methods to create ensembles that can credibly predict probabilities. Because observational errors can be diagnosed fairly well, and the updating process used in standard data assimilation methods is reasonably well founded, the characteristics of the ensemble forecast are largely determined by model uncertainty. In applying this technique to real models, it is important that the data assimilation technique matches the observations with typical trajectories of the climate model, rather than using unphysical transients. Cotter [22] shows how this can be achieved in a simple system by defining a projection onto the slow manifold in terms of an explicit map.

Rougier [23] discusses the same ideas in the more general context of inherently stochastic models with uncertain static parameters. It is noted that, in the environmental sciences, initial-condition sensitivity and attracting sets pose a major research challenge, which is increased by the existence of stochasticity and parametric uncertainty. The task of performing data assimilation with uncertain static parameters is labelled ‘intractable and unsolved’. However, the paper concludes by warning against what appears to be a counsel of despair. Although off-the-shelf methods cannot be expected to work when there are complicating factors like stochasticity, we may be able to find success by applying physical insights to tune the data assimilation method.

Of all the available methods for processing the results, it is important to understand which methods give the most useful predictions. Chandler [24] presents and analyses a statistical framework for combining projections of future climate from different climate simulators. Information from individual simulators is automatically weighted, alongside that from historical observations and from prior knowledge. The framework demonstrates that some subjective judgements are inevitable when interpreting multiple climate change projections. By clarifying precisely what these judgements are, it provides increased transparency in the ensuing analyses.

Observational data should, in principle, provide guidance on trends in local climate at the specific thresholds relevant to particular impact or policy endeavours. After noting that global temperature rises are largely irrelevant for local impact assessments, Chapman *et al.* [25] show how this guidance can be achieved for local climate trends. The level of detail needed from climate models, if they are to be used as tools to assess climate change impact, is quantified.

## 4. Future research directions

While much has been achieved with simple models with regard to elucidating mechanisms and simulating features of the observed climate, in many cases, it is not yet clear to what extent simple models have predictive value. The next steps include establishing such value, if at all possible. This can only be achieved through further progress in identifying the physical principles that control the overall behaviour of the climate system, assuming such principles exist. The simple models are already playing a useful role in showing how particular physical assumptions can translate into solutions that match aspects of the observed climate record. However, for predictive applications, it is essential that simple models can also be shown to respond correctly to perturbations.

The studies in this Theme Issue of the behaviour of complex models are a small selection of work being undertaken across the whole range of GCM components. The detail provided by GCMs is required to make quantitative predictions of use to policy makers. However, there are gaps that need to be filled before we can be confident that GCMs are well founded and that the uncertainties are well represented. The need to resolve a huge range of scales means that there is a serious problem in validating the predictions of the overall behaviour of the climate system. This is where simple models can be helpful.

In simple models, the exercise of fitting the models to observational data is building up considerable expertise and represents an exciting opportunity to inform stochastic schemes for representing small-scale processes in GCMs. Representing sub-grid-scale fluctuations by stochastic parametrizations (and improving deterministic physics parametrizations) in the GCM of the European Centre for Medium-range Weather Forecasts has been shown by Berner *et al.* [26] to decrease the systematic bias of the Northern Hemispheric circulation, reduce the precipitation bias in the tropics, and improve the characteristics of convectively coupled waves and tropical variability. In other recent examples, Plant [27] has studied the time-dependent and stochastic modelling of atmospheric convective systems as a collection of distinct plumes, and Williams [28] has studied the climatic impacts of stochastic fluctuations in air–sea fluxes. In complex models, the use of data assimilation methods in developing ensembles that can properly quantify uncertainties is a rapidly developing area that can make major contributions to the advice given to the climate community.

A major future need is the identification of the physical principles that control the overall behaviour of the climate system and that would make reliable prediction possible. For example, while we know that an energy budget can be constructed for each part of the Earth system, in that the energy change is explained by changes in the exchanges of energy with other components, it is not known what controls the average radiative equilibrium temperature of the Earth as viewed from space. It may be that there are no such principles, in which case, there are extra uncertainties. Mathematics can only apply the principles, not invent them. However, mathematics can at least be used to evaluate the consequences of physical theories and match them with reality.

Many pressing challenges in climate science require closer collaboration between climate scientists on the one hand, and mathematicians and statisticians on the other hand. The flow of information between these two parties should be two way. For example, climate science needs to embrace advanced statistical and stochastic modelling, in order to continue to advance beyond the deterministic paradigm embodied in traditional climate models. At the same time, the mathematical and statistical sciences need to embrace the exciting challenges being driven by new developments in climate science, such as the construction of emulators capable of simulating complex GCMs, and the estimation of future climate and its uncertainties from ensembles of such models.

It is fitting that this Theme Issue appears in the same year that has been designated as a special year for the Mathematics of Planet Earth (http://mpe2013.org). Dozens of international scientific societies, universities, research institutes and foundations have come together to organize a year-long series of programmes, summer schools, workshops, public lectures and exhibitions devoted to this topic. The initiative enjoys the patronage of UNESCO and is endorsed by the International Mathematical Union and the International Council of Industrial and Applied Mathematics. Although this Theme Issue is not formally affiliated with Mathematics of Planet Earth 2013, we hope that the papers contained in it will act as inspiration for the discussions and for setting future research directions.
